# Development and prospective multicenter evaluation of the long noncoding RNA MALAT-1 as a diagnostic urinary biomarker for prostate cancer

**DOI:** 10.18632/oncotarget.2691

**Published:** 2014-11-04

**Authors:** Fubo Wang, Shancheng Ren, Rui Chen, Ji Lu, Xiaolei Shi, Yasheng Zhu, Wei Zhang, Taile Jing, Chao Zhang, Jian Shen, Chuanliang Xu, Huiqing Wang, Haifeng Wang, Yang Wang, Bin Liu, Yaoming Li, Ziyu Fang, Fei Guo, Meng Qiao, Chengyao Wu, Qiang Wei, Danfeng Xu, Dan Shen, Xin Lu, Xu Gao, Jianguo Hou, Yinghao Sun

**Affiliations:** ^1^ Department of Urology, Shanghai Changhai Hospital, Second Military Medical University, Shanghai, China; ^2^ Department of Urology, Changshu NO. 2 People's Hospital, Changshu, Jiangsu Province, China; ^3^ Department of Pathology, Shanghai Changhai Hospital, Second Military Medical University, Shanghai, China; ^4^ Department of Urology, West China Hospital, Sichuan University, Chengdu, Sichuan, China; ^5^ Department of Urology, Shanghai Changzheng Hospital, Second Military Medical University, Shanghai, China

**Keywords:** prostate cancer, urine, biomarker, prostate biopsy, PSA, MALAT-1

## Abstract

The current strategy for diagnosing prostate cancer (PCa) is mainly based on the serum prostate-specific antigen (PSA) test. However, PSA has low specificity and has led to numerous unnecessary biopsies. We evaluated the effectiveness of urinary metastasis-associated lung adenocarcinoma transcript 1 (MALAT-1), a long noncoding RNA, for predicting the risk of PCa before biopsy. The MALAT-1 score was tested in a discovery phase and a multi-center validation phase. The predictive power of the MALAT-1 score was evaluated by the area under receiver operating characteristic (ROC) curve (AUC) and by decision curve analysis. As an independent predictor of PCa, the MALAT-1 score was significantly higher in men with a positive biopsy than in those with a negative biopsy. The ROC analysis showed a higher AUC for the MALAT-1 score (0.670 and 0.742) vs. the total PSA (0.545 and 0.601) and percent free PSA (0.622 and 0.627) in patients with PSA values of 4.0-10 ng/ml. According to the decision curve analysis, using a probability threshold of 25%, the MALAT-1 model would prevent 30.2%-46.5% of unnecessary biopsies in PSA 4–10 ng/ml cohorts, without missing any high-grade cancers. Our results demonstrate that urine MALAT-1 is a promising biomarker for predicting prostate cancer risk.

## INTRODUCTION

Prostate cancer (PCa) is the most commonly diagnosed cancer in men in the United States, with an estimated 238,599 new cases and 29,720 deaths in the year 2013 [1]. Its global incidence is on the rise, especially in Asian countries [2, 3]. Once PCa invades local organs or spreads distantly, only palliative treatments can be offered. Patients with early stage PCa can survive more than 10 years after diagnosis. Thus, to reduce PCa-related mortality, efforts are being devoted to increase the detection rate of PCa at an early stage. The wide use of prostate-specific antigen (PSA) has greatly improved the early diagnosis of clinically localized PCa [4], resulting in a signif icant decrease in PCa-specific death [5]. However, as PSA is organ-specific rather than tumor-specific, elevated PSA is also associated with other conditions, such as prostatitis, benign prostatic hyperplasia and recent ejaculation [6]. Nevertheless, men with elevated serum PSA are recommended to undergo a biopsy for a definitive diagnosis. However, PSA has a low specificity of 25–40% in the so-called “diagnostic grey zone” (PSA 4–10 ng/ml), which has also resulted in many unnecessary biopsies and biopsy-related financial, social and psychological burdens. Therefore, there is an urgent need to develop more sensitive, specific biomarkers to diagnose PCa at an early stage while avoiding unnecessary biopsies. A wide range of promising PCa biomarkers has been reported, including CpG hypermethylation of GSTP1 [7], TMPRSS2:ERG gene fusion [8], AMACR [9], sarcosine [10], and the long noncoding RNA urine biomarker prostate cancer gene 3 (PCA3) [11]. Currently, PCA3 is the most extensively studied urine biomarker. PCA3 is a prostate-speciﬁc non-coding RNA that is highly overexpressed in PCa compared to the normal prostate [12, 13]. As prostate cells can be detected in the urine of men after a digital rectal examination (DRE), urine-based diagnostic tests have the advantage of being non-invasive or minimally invasive. The Progensa PCA3 test has been approved by the US Food and Drug Administration and is commercially available to guide repeat biopsy decision-making.

Metastasis-associated lung adenocarcinoma transcript 1 (MALAT-1) has been described as a regulator of metastasis and motility, and its expression is associated with metastasis in non-small cell lung cancer. It is a noncoding RNA of more than 8,000 nt derived from chromosome 11q13 [14]. MALAT-1 is overexpressed in multiple types of human malignancies, including hepatocellular cell carcinoma, lung adenocarcinoma, endometrial stromal sarcoma, and colorectal cancer [15-18]. Using RNA-Seq and quantitative RT-PCR, we found that MALAT-1 is upregulated in prostate cancer tissues compared with adjacent normal tissues and benign prostatic hyperplasia (BPH) tissues [19]. Higher MALAT-1 expression is correlated with aggressive characteristics of PCa in prostate cancer tissue. MALAT-1 silencing dramatically inhibited PCa cell growth, invasion and migration and induced cell cycle arrest *in vitro* and *in vivo*. Furthermore, we showed that a circulating MALAT-1 fragment (MD-miniRNA) outperformed PSA in predicting prostate biopsy outcomes, suggesting that MALAT-1 may be a promising biomarker for diagnosing PCa.

In this study, we investigated the potential diagnostic efficacy of urinary MALAT-1 transcript in a retrospective discovery phase (n=218) and a prospective multicenter cohort (n=216). We also evaluated the potential of the MALAT-1 score and the MALAT-1 model for PCa diagnosis.

## RESULTS

Initially, 536 patients were included in this study, from which 29 samples were excluded for insufficient RNA extraction from the sediments. After quantitative RT-PCR analysis, another 73 patients were excluded, as their PSA Ct value was above 28 [20], indicating insufficient prostate cell collection. Therefore, 434 patients (218 patients in the discovery phase and 216 patients in the validation phase) were finally recruited in this study. The clinical and pathological data are listed in Table 1. PCa-associated risk factors (age, total PSA (tPSA), volume, percent free PSA (%fPSA) and DRE) were all significantly higher in patients with a positive biopsy compared with those with a negative biopsy in all the patients in the discovery phase. However, tPSA failed to discriminate positive biopsy results from negative biopsy results in the PSA 4-10 ng/ml cohort. These results were confirmed in the validation phase.

**Table 1:** Prostate cancer associated risk factors in both discovery and validation cohort

	Discovery Phase								Validation Phase							
Parameter	Overall Cohort				PSA 4-10ng/ml Cohort				Overall Cohort				PSA 4-10ng/ml Cohort			
	Entire	Negative	Positive	*p* value	Entire	Negative	Positive	*p* value	Entire	Negative	Positive	*p* value	Entire	Negative	Positive	*p* value
Age, yr				0.041*				0.244*				0.990*				0.891*
No. pts (%)	218(100.0)	133(61.0)	85(39.0)		94(100.0)	71(75.5)	23(24.5)		216(100.0)	135(62.5)	81(37.5)		89(100.0)	63(70.8)	26(29.2)	
Mean	65.8	64.1	68.3		64.2	63.3	67.1		65.3	64.1	67.4		63.8	62.9	65.9	
SD	7.5	7.9	6.0		8.6	8.9	6.8		7.3	7.4	6.5		7.8	8.0	6.8	
tPSA, ng/ml				<0.001^#^				0.521^#^				<0.001^#^				0.134^#^
No. pts (%)	218(100.0)	133(61.0)	85(39.0)		94(100.0)	71(75.5)	23(24.5)		216(100.0)	135(62.5)	81(37.5)		89(100.0)	63(70.8)	26(29.2)	
Median	10.8	9.3	14.5		6.6	6.5	6.8		10.8	10.1	17.1		7.4	7.2	8.1	
IQR	6.7-16.1	5.9-12.5	9.5-41.5		5.5-8.7	5.5-8.6	5.6-9.0		7.8-19.6	7.2-14.9	8.9-31.2		6.1-8.6	6.0-8.4	6.3-9.0	
Volume, ml^¶^				0.007^#^				0.001^#^				<0.001^#^				0.019^#^
No. pts (%)	218(100.0)	133(61.0)	85(39.0)		94(100.0)	71(75.5)	23(24.5)		216(100.0)	135(62.5)	81(37.5)		89(100.0)	63(70.8)	26(29.2)	
Median	43.4	50.3	42.3		50.4	54.3	29.4		44.8	50.3	40.7		44.7	50.5	39.9	
IQR	32.6-63.2	33.2-68.0	29.2-49.1		32.1-68.4	38.7-71.0	25.1-48.8		34.9-67.6	38.2-81.4	29.3-54.0		34.9-72.4	38.2-78.3	29.5-48.4	
%fPSA				0.001^#^				0.081^#^				<0.001^#^				0.06^#^
No. pts (%)	193(100.0)	122(63.2)	71(36.8)		94(100.0)	71(75.5)	23(24.5)		195(100.0)	124(63.6)	71(36.4)		89(100.0)	63(70.8)	26(29.2)	
Median	0.15	0.16	0.14		0.15	0.17	0.15		0.15	0.18	0.12		0.16	0.17	0.15	
IQR	0.11-0.21	0.12-0.23	0.08-0.18		0.12-0.22	0.12-0.24	0.11-0.18		0.11-0.22	0.13-0.26	0.07-0.18		0.12-0.21	0.13-0.22	0.08-0.18	
Suspicious DRE				<0.001^§^				0.098^§^				<0.001^§^				0.006^§^
No. pts	218	133	85		94	71	23		216	135	81		89	63	26	
No. %	63(28.9)	27(21.1)	36(41.4)		22(23.4)	13(21.3)	9(39.1)		58(26.9)	23(17.0)	39(43.2)		18(20.2)	8(12.7)	10(38.5)	
MALAT-1 Score				<0.001^#^				0.001^#^				<0.001^#^				0.012^#^
No. pts (%)	218(100.0)	133(61.0)	85(39.0)		94(100.0)	71(75.5)	23(24.5)		216(100.0)	135(62.5)	81(37.5)		89(100.0)	63(70.8)	26(29.2)	
Median	92.8	65.4	155.7		92.7	75.0	155.7		100.1	77.5	160.3		83.9	75.2	167.6	
IQR	32.9-185.9	24.1-139.0	67.5-258.3		33.0-162.9	26.8-150.7	113.2-296.0		49.4-203.5	46.9-163.2	79.2-270.9		50.5-177.5	48.7-144.5	71.5-225.0	
Biopsy Gleason																
sum, no. (%)																
≤6			22(25.9)				8(34.8)				24(29.6)				11(42.3)	
7			39(45.9)				10(43.5)				28(34.6)				13(50.0)	
≥8			24(28.2)				5(21.7)				29(35.8)				2(7.7)	

Yr= years; PSA = prostate-specific antigen; SD = standard deviation; IQR = interquartile range; tPSA = total PSA; PSAD = PSA density (serum PSA/prostate volume); %fPSA = percent free PSA; DRE = digital rectal examination; MALAT-1 = metastasis associated in lung adenocarcinoma transcript 1.

¶ Estimated by transrectal ultrasound.

* Student't Test.

# Mann-Whitney U test.

§ Pearson Chi-square Test.

**Figure 1:**
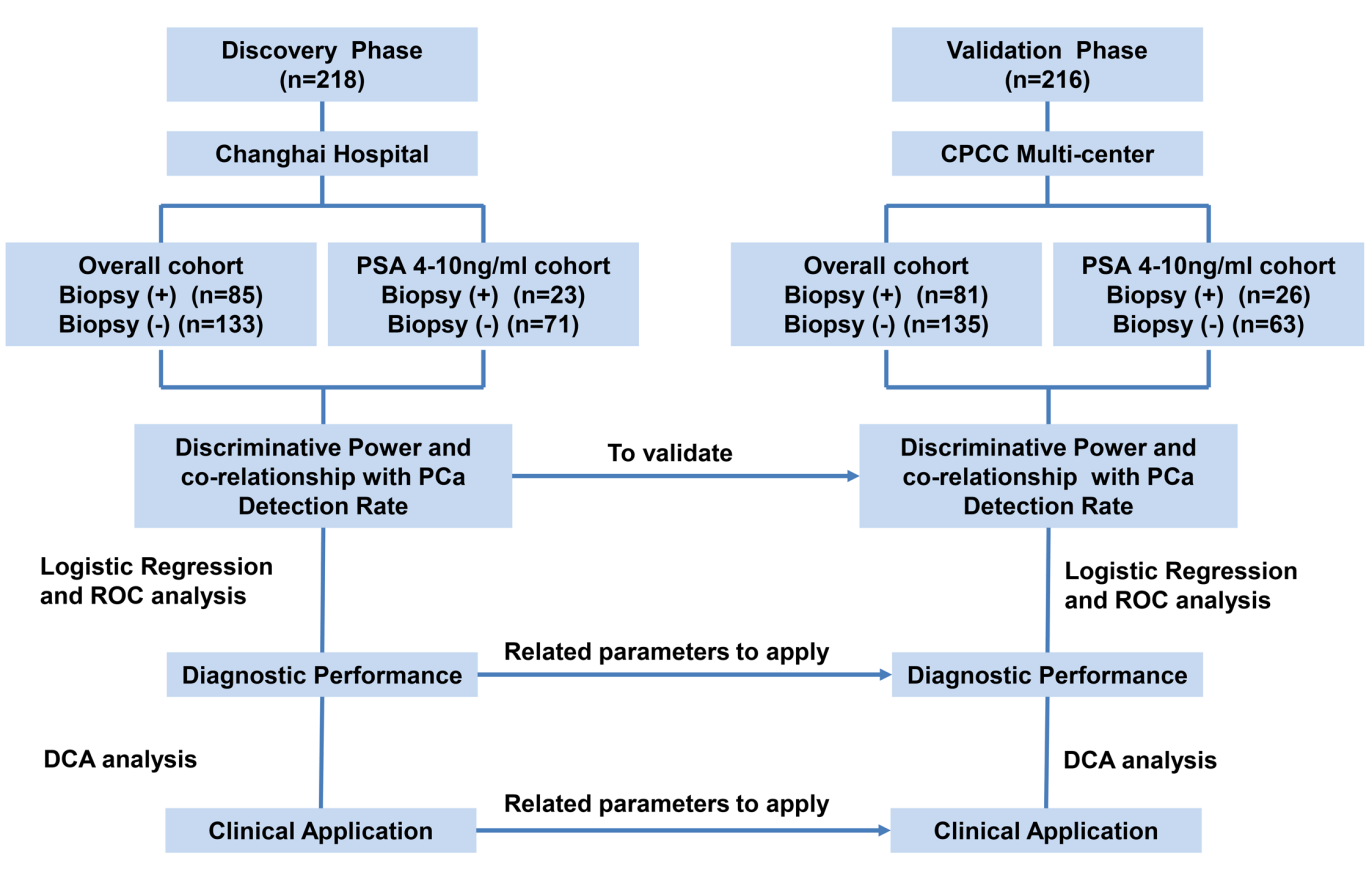
Study design CPCC=Chinese Prostate Cancer Consortium; PSA=prostate-specific antigen; ROC=receiver operating characteristic curve; DCA=decision curve analysis.

### MALAT-1 score sufficed to differentiate positive from negative prostate biopsy results and strongly correlated with the PCa detection rate

**Figure 2:**
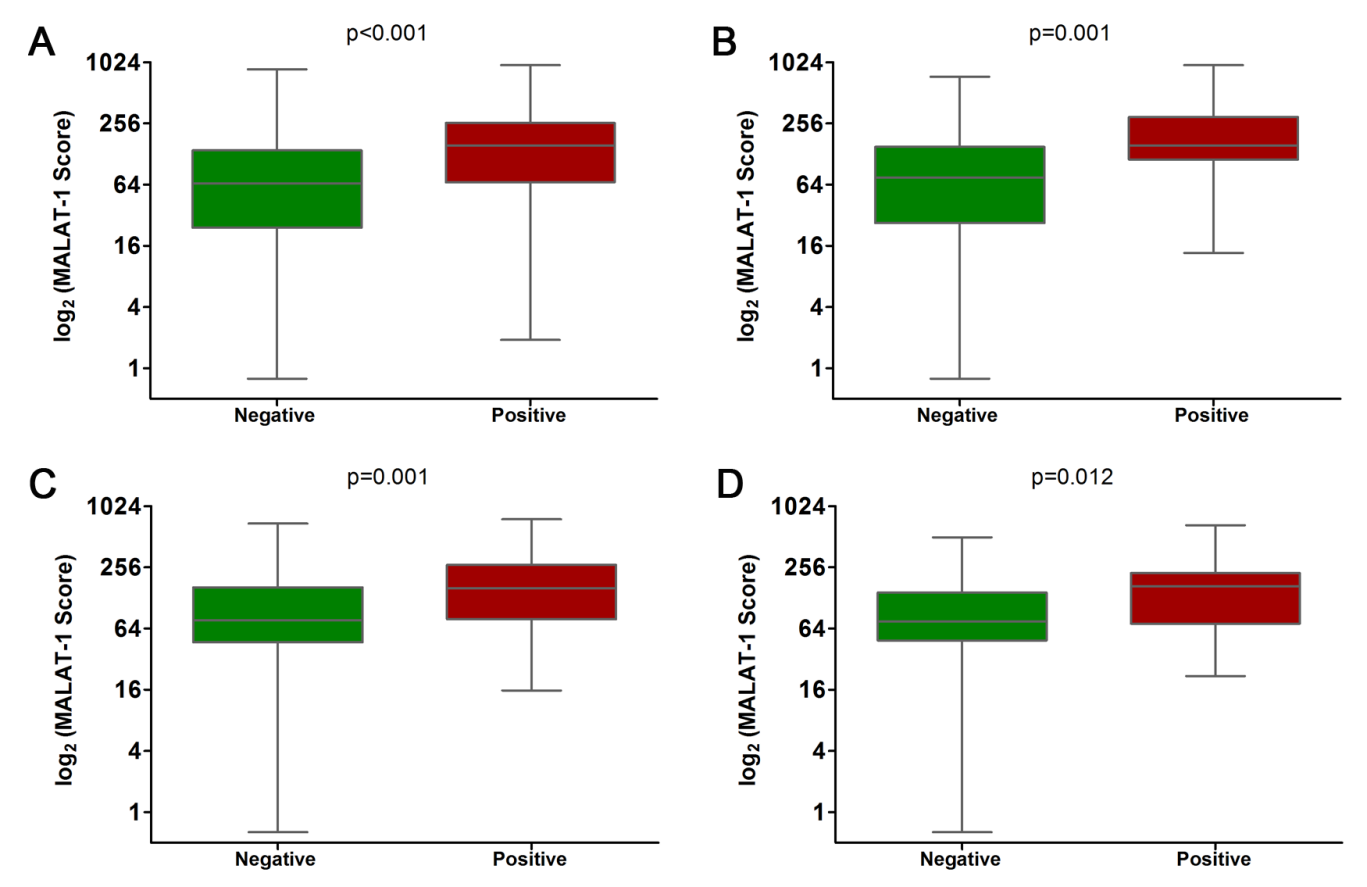
Comparison of MALAT-1 score between positive and negative biopsies in the discovery and validation phases (A) Overall cohort in the discovery phase. (B) PSA 4-10 ng/ml cohort in the discovery phase. (C) Overall cohort in the validation phase. (D) PSA 4-10 ng/ml cohort in the validation phase.

To explore whether the MALAT-1 score could be a useful biomarker to diagnose PCa in all patients and in the PSA 4-10 ng/ml cohort (which is of special clinical interest), the MALAT-1 score was tested in the discovery phase and evaluated in the validation phase. Our results revealed that the MALAT-1 score was significantly higher in positive biopsy patients in both the overall (Figure 2A) and the PSA 4-10 ng/ml cohorts (Figure 2B). The results were confirmed in the validation phase (Figure 2C for the overall cohort and Figure 2D for the PSA 4-10 ng/ml cohort).

**Figure 3:**
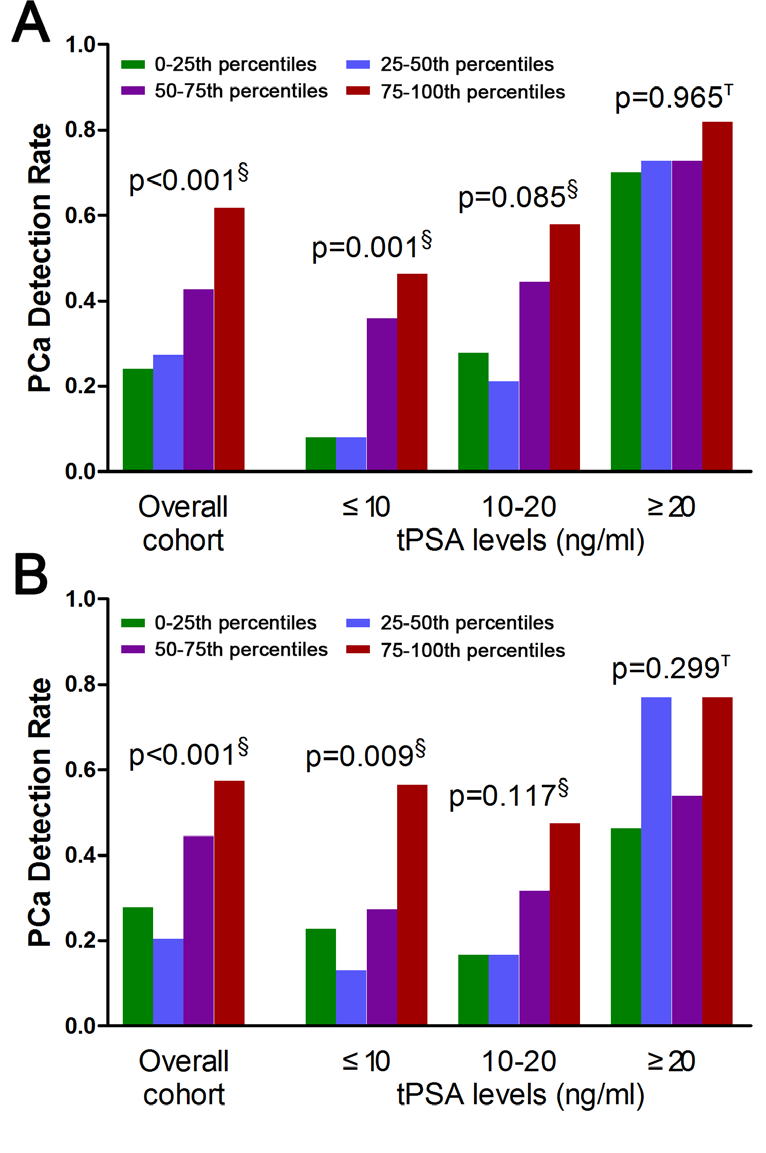
Prostate cancer detection rate in subjects with low (green), intermediate (blue), high (purple) and very high (red) MALAT-1 scores in the overall cohort and in subgroups based on PSA levels (A) Discovery phase. (B) Validation phase. ^§^Pearson chi-square. ^Т^Fisher's exact test. PSA=prostate-specific antigen.

Correlation analysis demonstrated that the MALAT-1 score did not correlate with other risk factors or the Gleason score (Supplemental Table 1). In addition, we evaluated the discriminative power of the MALAT-1 score in the overall cohort as well as in subsets of patients based on their PSA levels. In the overall cohort, the detection rate of PCa increased significantly with the MALAT-1 score: 24.1%, 27.3%, 42.6%, and 61.8% for men with low (0-25th percentile), intermediate (25th-50th percentile), high (50th-75th percentile), and very high (75th-100th percentile) MALAT-1 scores, respectively (Figure 3A, p<0.001). The association of increasing PCa detection rates with a higher MALAT-1 score was consistently observed in the PSA 4-10 ng/ml cohort (Figure 3A, p=0.001). In the validation phase, the same results were obtained and are shown in Figure 3B. The detection rate of PCa increased with the MALAT-1 score in the PSA >10 ng/ml cohort, but the increments did not meet significance in the discovery (p=0.085 for PSA 10-20 ng/ml and p=0.965 for PSA >20 ng/ml) or validation phases (p=0.117 for PSA 10-20 ng/ml and p=0.299 for PSA >20 ng/ml). Therefore, our results demonstrated that the MALAT-1 score was strongly correlated with prostate cancer risk in the overall group as well as in the subgroup with PSA=4-10 ng/ml and may serve as a noninvasive biomarker for the detection of PCa in urine.

### Logistic regression evaluation of diagnosis performance of the MALAT-1 score in the discovery phase and applied in the validation phase

**Figure 4:**
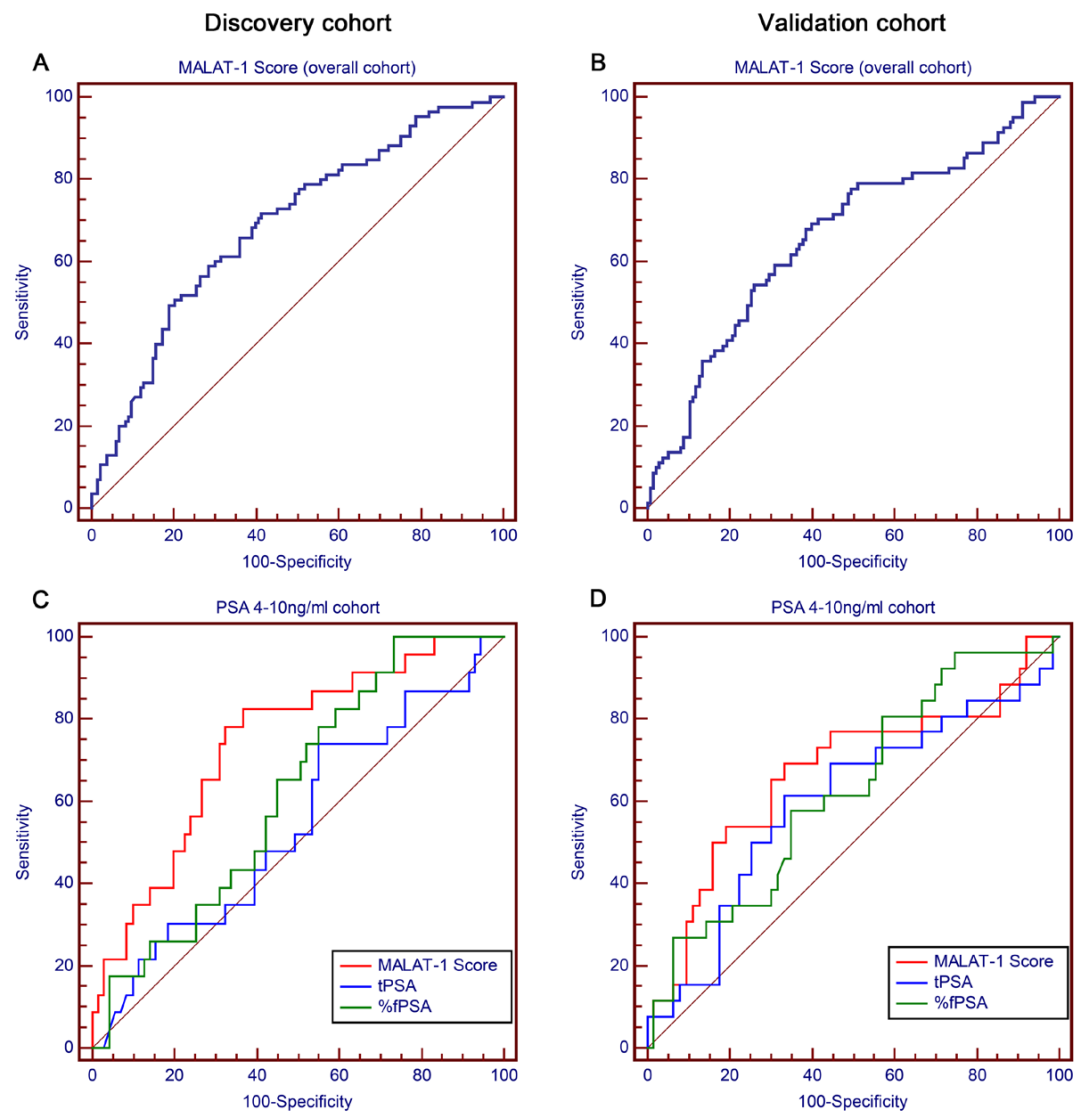
Receiver operating characteristic curve analysis for evaluating the diagnostic performance of the MALAT-1 score Area under the curve (AUC) estimation for the MALAT-1 score in the overall cohort in the (A) discovery phase and (B) validation phase. Comparison of the diagnostic performance of the MALAT-1 score, tPSA and %fPSA in the PSA 4-10 ng/ml cohort in the (C) discovery phase and (D) validation phase.

In the discovery phase, in univariable and multivariable logistic regression models, MALAT-1 score, age, tPSA, volume, %fPSA and DRE were independent risk factors in the overall cohort, while tPSA was excluded in the PSA grey zone cohort (Tables 2 and 3, Supplemental Figure 1). In univariable logistic regression models, the MALAT-1 score in the overall cohort represented a comparable informative parameter in the prediction of PCa (AUC: 0.688; Figure 4A) to serum PSA (AUC: 0.721) and was superior to serum PSA in the PSA grey zone cohort (MALAT-1 score AUC: 0.742; tPSA AUC: 0.545; Figure 4C) (Supplemental Table 2). There was a trend in the overall and PSA grey zone cohorts that the MALAT-1 score was superior to %fPSA (AUC: 0.639, 0.622), but it was not statistically significant. Each model's predictive accuracy (PA) and the AUC are displayed in Table 3. The MALAT-1 score-based model demonstrated a higher AUC of 0.840 and PA of 77.20% in the prediction of PCa and resulted in an increased AUC of 0.0167 and increased PA of 2.59% in the overall cohort (Supplemental Figure 2). In the PSA grey zone cohort, the MALAT-1 score-based model demonstrated a higher AUC of 0.853 and PA of 79.79% in the prediction of PCa and resulted in an increased AUC of 0.0318 and increased PA of 5.32%.

**Table 2:** Univariable logistic regression models predicting prostate cancer

	Discovery Phase				Validation Phase			
Variables	Overall Cohort		PSA 4-10ng/ml Cohort		Overall Cohort		PSA 4-10ng/ml Cohort	
	OR (95% CI); *p*	AUC (95% CI)	OR (95% CI); *p*	AUC (95% CI)	OR (95% CI); *p*	AUC (95% CI)	OR (95% CI); *p*	AUC (95% CI)
MALAT-1	1.0030(1.0013-1.0047);	0.688	1.0041(1.0013-1.0069);	0.742	1.0036(1.0016-1.0056);	0.661	1.0045(1.0007-1.0083);	0.670
Score	<0.001	(0.622-0.749)	0.0043	(0.641 to 0.826)	<0.001	(0.594-0.724)	0.0199	(0.562-0.766)
Age	1.0913(1.0444-1.1404); <0.001	0.658 (0.591-0.721)	1.0635(0.9959-1.1357); 0.0662	0.610 (0.504-0.709)	1.0717(1.0265-1.1189); 0.0016	0.625 (0.557-0.690)	1.0566(0.9893-1.1285); 0.1012	0.600 (0.491-0.703)
tPSA	1.0644(1.0335-1.0962); <0.001	0.721 (0.656-0.779)	1.0723(0.8148-1.4111); 0.6183	0.545 (0.439-0.648)	1.0518(1.0272-1.0770); <0.001	0.680 (0.614-0.742)	1.2414(0.9165-1.6815); 0.1624	0.601 (0.492-0.704)
Volume	0.9815(0.9685-0.9946); 0.0059	0.608 (0.539-0.673)	0.9588(0.9318-0.9865); 0.0038	0.738 (0.637-0.823)	0.9736(0.9605-0.9869); <0.001	0.661 (0.593-0.724)	0.9737(0.9513-0.9966); 0.0248	0.659 (0.551-0.756)
%fPSA	0.0007(0.0000-0.0481); <0.001	0.639 (0.567-0.707)	0.0003(0.0000-0.6268); 0.0374	0.622 (0.516-0.720)	0.0003(0.0000-0.0183); <0.001	0.689 (0.619-0.753)	0.0007(0.0000-1.2247); 0.0566	0.627 0.518 to 0.728
DRE	2.8804(1.5782-5.2715); <0.001	0.610 (0.542-0.675)	2.8681(1.0229-8.0420); 0.0452	0.604 (0.498-0.704)	3.7051(1.9769-6.9440); <0.001	0.631 (0.563-0.695)	4.2969(1.4539-12.6992) 0.0084	0.629 (0.520-0.729)

PSA = prostate-specific antigen; OR = odds ratio; CI = confidence interval; AUC = area under the receiver operating characteristic curve; MALAT-1 = metastasis associated in lung adenocarcinoma transcript 1; tPSA = total PSA; %fPSA = percent free PSA; DRE = digital rectal examination.

**Table 3:** Multivariable logistic regression models predicting prostate cancer

	Discovery Phase				Validation Phase			
	Overall Cohort		PSA 4-10ng/ml Cohort		Overall Cohort		PSA 4-10ng/ml Cohort	
Variables	Base model‡	Base model plus MALAT-1 Score	Base model∮	Base model plus MALAT-1 Score	Base model‡	Base model plus MALAT-1 Score	Base model∮	Base model plus MALAT-1 Score
	OR (95% CI); *p*	OR (95% CI); *p*	OR (95% CI); *p*	OR (95% CI); *p*	OR (95% CI); *p*	OR (95% CI); *p*	OR (95% CI); *p*	OR (95% CI); *p*
MALAT-1 Score	-	1.0026(1.0006-1.0047); 0.0099	-	1.0032(1.0003-1.0062); 0.0310	-	1.0031(1.0006-1.0055); 0.0131	-	1.0063(1.0008-1.0117); 0.0239
Age	1.0967(1.0383-1.1583); 0.0009	1.0889(1.0297-1.1515); 0.0028	1.1210(1.0336-1.2158); 0.0058	1.1048(1.0162-1.2011); 0.0195	1.0905(1.0326-1.1517); 0.0019	1.0870(1.0286-1.1487); 0.0031	1.0936(1.0127-1.1810); 0.0226	1.1186(1.0228-1.2233); 0.0142
tPSA	1.0511(1.0188-1.0844); 0.0017	1.0506(1.0181-1.0841); 0.0021	1.2792(0.8942-1.8298); 0.1777	1.3029(0.8966-1.8934); 0.1653	1.0381(1.0105-1.0665); 0.0066	1.0353(1.0084-1.0630); 0.0099	1.3000(0.9280-1.8212); 0.1271	1.2376(0.8705-1.7595); 0.2350
Volume	0.9816(0.9656-0.9978); 0.0261	0.9817(0.9653-0.9985); 0.0324	0.9660(0.9378-0.9950); 0.0220	0.9704(0.9417-0.9999); 0.0496	0.9753(0.9592-0.9918); 0.0034	0.9759(0.9594-0.9927); 0.0050	0.9724(0.9461-0.9994); 0.0450	0.9707(0.9430-0.9993); 0.0444
%fPSA	0.0006(0.0000 to 0.1078); 0.0050	0.0012(0.0000-0.2464); 0.0132	0.0000(0.0000-0.3282); 0.0280	0.0001(0.0000-0.8535); 0.0464	0.0040(0.0000-0.4447); 0.0216	0.0019(0.0000-0.2415); 0.0112	0.0008(0.0000-5.4886); 0.1132	0.0000(0.0000-0.9040); 0.0478
DRE	2.6254(1.2299-5.6042); 0.0126	2.7310(1.2575-5.9312); 0.0111	3.3824(0.9338-12.2515); 0.0635	3.7679(1.0014-14.1779); 0.0498	2.8602(1.3328-6.1380); 0.0070	2.7928(1.2784-6.1013); 0.0100	4.6171(1.3434-15.8683); 0.0152	4.0829(1.0674-15.6179); 0.0399
PA (%)	74.61	77.20	74.47	79.79	77.44	80.00	71.91	76.40
Increment PA (%)	-	2.59	-	5.32	-	2.56	-	4.49
AUC (95%CI)	0.815 (0.753-0.867)	0.840(0.781-0.889)	0.821(0.729-0.893)	0.853(0.765-0.918)	0.817(0.755-0.869)	0.833(0.773-0.882)	0.772(0.671-0.855)	0.799(0.701-0.877)
Increment AUC (95%CI)	-	0.0167(−0.00853-0.0419)	-	0.0318(−0.0458 to 0.109)	-	0.0156(−0.0120-0.0432)	-	0.0269(−0.0373-0.0910)

PSA = prostate-specific antigen; MALAT-1 = metastasis associated in lung adenocarcinoma transcript 1; OR = odds ratio; CI = confidence interval; tPSA = total PSA; %fPSA = percent free PSA; DRE = digital rectal examination; PA = Predictive accuracy; AUC = area under the curve.

‡ The base model consisted of age, tPSA, volume, %fPSA and DRE.

∮ The base model consisted of age, volume, %fPSA and DRE.

The parameters estimated from the discovery phase were evaluated in the independent validation phase. In the univariable and multivariable logistic regression models, comparable independent risk factors were obtained in the overall and PSA grey zone cohorts. There was also a trend in the overall and PSA grey zone cohorts that the MALAT-1 score (AUC: 0.661; Figure 4B) was comparable to serum tPSA (AUC: 0.680) and %fPSA (AUC: 0.689), and the MALAT-1 score (AUC: 0.670) was superior to serum tPSA (AUC: 0.601) and %fPSA (AUC: 0.627) in the PSA grey zone (Figure 4D) (Table 2). Applying this model to the validation models, the MALAT-1 score-based model demonstrated a higher AUC of 0.833 and PA of 80.00% in the prediction of PCa and resulted in an increment AUC of 0.0156 and increase of PA of 2.56% in the overall cohort. In the PSA grey zone cohort, the MALAT-1 score-based model demonstrated a higher AUC of 0.799 and PA of 76.40% in the prediction of PCa and resulted in an increased AUC of 0.0269 and increased PA of 4.49% (Table 3).

### Decision curve analysis evaluation of the accuracy of the diagnostic model with and without MALAT-1

As the diagnosis of prostate cancer in the PSA grey zone is of particular interest, the decision curve analysis focused on this group of patients, while the DCAs for the overall cohorts are reported in the supplemental data (Supplemental Figure 3 and Supplemental Tables 3, 4).

**Figure 5:**
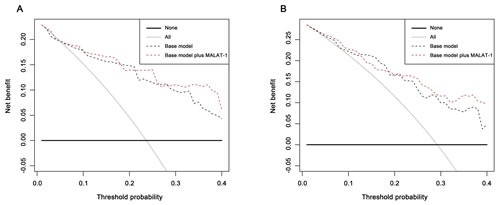
Decision curve analysis for positive biopsy prediction in the PSA 4-10 ng/ml cohorts by the base model (base model contains age, volume, %fPSA and DRE) in the (A) discovery phase and (B) validation phase The dashed black line indicates the base model that contains age, volume, %fPSA and DRE; the solid black line shows the prediction model that includes only age, PSA level, DRE result, and prostate volume. The horizontal line along the *x*-axis assumes that no patient will have PCa (no patient should undergo a prostate biopsy), whereas the solid gray line assumes that all patients will have PCa (all patients will need to undergo a prostate biopsy).

As the decision curve indicates, in the grey zone cohort discovery phase, the base model plus MALAT-1 was superior to the base model, with a higher net benefit for almost all threshold probabilities >10% (Figure 5A, Table 4). For the patients with PSA values of 4.0-10.0 ng/ml in the validation phase, the base model plus MALT-1 was better than the base model for all threshold probabilities >22% (Figure 5B).

**Table 4:** Net benefit and reduction in avoidable biopsies for the base model and base model plus MALAT-1 compared to the ‘treat all’ strategy to biopsy every patient for different threshold probabilities in the same range for patients of PSA diagnostic “grey zone” (PSA 4.0-10.0ng/ml)

	Threshold probability (%)		10	15	20	25	30	35	40
Discovery Phase	Net benefit	Base model∮	17.921	15.623	14.785	11.470	9.831	7.610	4.301
		Base model∮+MALAT-1	17.443	16.572	13.978	13.978	11.060	10.918	6.093
		Treat all	15.173	10.183	4.570	−1.792	−9.063	−17.452	−27.240
	Net reduction in avoidable biopsies	Base model∮	24.731	30.824	40.860	39.785	44.086	46.544	47.312
		Base model∮+MALAT-1	20.430	36.201	37.634	47.312	46.953	52.688	50.000
Validation Phase	Net benefit	Base model∮	22.222	21.150	16.573	13.109	9.952	7.779	4.869
		Base model∮+MALAT-1	22.722	19.299	16.573	15.730	11.557	11.841	9.738
		Treat all	21.348	16.722	11.517	5.618	−1.124	−8.902	−17.978
	Net reduction in avoidable biopsies	Base model∮	7.865	25.094	20.225	22.472	25.843	30.979	34.270
		Base model∮+MALAT-1	12.360	14.607	20.225	30.337	29.588	38.523	41.573

MALAT-1 = metastasis associated in lung adenocarcinoma transcript 1. PSA = prostate-specific antigen; %fPSA = percent free PSA; DRE = digital rectal examination;

∮ The base model consisted of age, volume, %fPSA and DRE.

In the grey zone cohort of the discovery phase, the base model plus MALAT-1 detected more cancer than the base model at a threshold of 25% (13.97% vs. 11.47%) (Table 4). At this threshold, the base model plus MALAT-1 also prevented more unnecessary biopsies than the base model (47.32% vs. 39.78%) (Table 5). At the threshold of 25%, the base model with MALAT-1 could spare 8.5% more biopsies (46.5% vs. 38.0%), both at the risk of missing only one (4.3%) cancer patient in the discovery phase.

**Table 5:** Number of total and high-grade (defined as Gleason ≧ 7) PCa missed and reduction in biopsies according to threshold probability in the range of 10-40% for the Base model and Base model plus MALAT-1 for patients of the overall cohort

	Probability cut-off, %	Model	PCa missed, No. (%)	High-grade PCa missed, No. (%)	Unnecessary Biopsies spared, No. (%)
Discovery Phase	15	Base model∮	1(4.3%)	0	21(29.6%)
		Base mode plus MALAT-1	1(4.3%)	0	25(35.2%)
	20	Base model∮	1(4.3%)	0	28(39.4%)
		Base mode plus MALAT-1	1(4.3%)	0	26(36.6%)
	25	Base model∮	1(4.3%)	0	27(38.0%)
		Base mode plus MALAT-1	1(4.3%)	0	33(46.5%)
	30	Base model∮	1(4.3%)	0	30(42.3%)
		Base mode plus MALAT-1	1(4.3%)	0	32(45.1%)
	35	Base model∮	1(4.3%)	0	32(45.1%)
		Base mode plus MALAT-1	1(4.3%)	0	36(50.7%)
	40	Base model∮	1(4.3%)	0	33(46.5%)
		Base mode plus MALAT-1	1(4.3%)	0	35(49.3%)
Validation Phase	15	Base model∮	1(3.8%)	1(3.8%)	15（23.8%）
		Base mode plus MALAT-1	0	0	9（14.3%）
	20	Base model∮	1(3.8%)	1(3.8%)	12（19.0%）
		Base mode plus MALAT-1	0	0	13（20.6%）
	25	Base model∮	1(3.8%)	1(3.8%)	13（20.6%）
		Base mode plus MALAT-1	0	0	19（30.2%）
	30	Base model∮	1(3.8%)	1(3.8%)	15（23.8%）
		Base mode plus MALAT-1	0	0	19（30.2%）
	35	Base model∮	1(3.8%)	1(3.8%)	19（30.2%）
		Base mode plus MALAT-1	1(3.8%)	1(3.8%)	23（36.5%）
	40	Base model∮	1(3.8%)	1(3.8%)	21（33.3%）
		Base mode plus MALAT-1	2(7.7%)	1(3.8%)	24（38.1%）

PCa = prostate cancer; MALAT-1 = metastasis associated in lung adenocarcinoma transcript 1; PSA = prostate-specific antigen; %fPSA = percent free PSA; DRE = digital rectal examination;

∮ The Base model consisted of age, volume, %fPSA and DRE.

In the validation phase, when the same threshold of 25% was applied, the base model plus MALAT-1 could also detect more cancers (15.73% vs. 13.11%) and prevented more unnecessary biopsies than the base model (30.33% vs. 22.47%). At the same threshold, the MALAT-1 based model could spare more unnecessary biopsies (30.2% vs. 20.6%) while missing fewer cancer patients (0 vs. 3.8%).

## DISCUSSION

The current standard approach to diagnose PCa is prostate biopsy, which is mostly based on elevated PSA and/or abnormal DRE. Although the introduction of PSA has greatly improved early PCa detection and stage migration as well as reduced PCa mortality [4], PSA has low specificity in discriminating between benign and malignant prostatic diseases, leading to unnecessary and/or repeat biopsies [21]. As a result, the expansion of PSA screening has led to intense debate as to overdiagnosis and, ultimately, overtreatment of low-risk patients. A recent report from the European Randomized Study of Screening for Prostate Cancer (ERSPC) and Prostate, Lung, Colorectal and Ovarian Cancer Screening Trial (PLCO) indicates a continued need for novel biomarkers to supplement serum PSA to accurately predict PCa risk and stratification of indolent and aggressive PCa [22, 23].

Although the incidence of PCa in China is reported to be 1.6/100,000 per year, its incidence has recently increased substantially, with an estimated incidence of 10/100,000 in 2010 compared with 1.71/100,000 in 1993 [24, 25]. With the implementation of more comprehensive screening programs in the developing cities and regions of China, the incidence rate may rapidly increase in the future, as evidenced by the fact that PCa ranks first among all types of urologic tumors and reaches 31.2/100,000 in Shanghai (2012) [26]. However, mass screening of PCa is not performed nationwide in China, especially in rural China, which results in a substantial portion of men who are diagnosed at an advanced stage with a high PSA level [27]. In our study, although the urine samples for the discovery and validation phases were collected from major hospitals in developed cities in China, the patients came from a variety of regions in China, including rural areas. As shown in Table 1, the PSA distribution of men who underwent prostate biopsy in both cohorts is dramatically different from those of western populations, with over 50% of men with a PSA>10 ng/ml. In contrast, with the implementation of PSA screening, over 90% of men referred for biopsy in western populations have PSA levels below 10 ng/ml. Apparently, the higher the PSA level is, the higher the risk of PCa. PSA is a good predictor when its level is >10 ng/ml. We compared the detection rate of prostate cancer in patients with different MALAT-1 scores in the PSA>10 ng/ml cohort. However, the differences between each group failed to meet the conventional levels of statistical significance. AUC-ROC analysis revealed that the diagnostic power of the MALAT-1 score (AUC in the discovery phase: 0.662; AUC in the validation phase: 0.640) was not superior to that of PSA (AUC in the discovery phase: 0.754; AUC in the validation phase: 0.785) in the PSA>10 ng/ml cohort in both the discovery (p=0.144, Z-test; Supplemental Figure 4A) and validation phases (p=0.022, Z-test; Supplemental Figure 4B). Thus, we particularly focused on evaluating the performance of the MALAT-1 score in patients with grey zone PSA, which is of great clinical interest.

Multiple derivatives, including the ratio of the free-to-total (f/t) PSA, PSA density or PSA velocity, have been developed to improve the diagnostic accuracy of PSA. %fPSA is most widely used in clinical settings to stratify the risk of PCa for men who have total PSA levels between 4 and 10 ng/ml [28]. In our data set, the diagnostic accuracy of the MALAT-1 score outperformed %fPSA. Applying a MALAT-1 cut-off value of 95 achieved better sensitivity and specificity than the recommended %fPSA cut-off of 0.16 in grey zone cohorts in both the discovery and validation phases (Table 6). Using a MALAT-1 score cut-off of 95 rather than the 0.16 cut-off of %fPSA could detect more cancers (19/23 vs. 15/23 in the discovery phase; 17/26 vs. 16/26 in the validation phase) and, at the same time, reduce unnecessary biopsies (44/71 vs. 39/71 in the discovery phase; 43/63 vs. 35/63 in the validation phase). Collectively, the MALAT-1 score may be more accurate than %fPSA, the most widely used parameter for PSA grey zone patients.

**Table 6:** Comparisons of the diagnostic performance of percent free PSA (%fPSA) and MALAT-1 Score at the recommended cut-off values in the PSA 4-10ng/ml cohorts in the discovery and validation phase

Study Phase	Variables	Cutoff	Sensitivity	95% CI	Specificity	95% CI	PCa Detected, %	Unnecessary Biopsies avoided, %
Discovery Phase	%fPSA	0.16	65.22	42.7 - 83.6	53.52	41.3 - 65.5	15/23,(65.2%)	39/71,(54.9%)
	MALAT-1 Score	95	82.61	61.2 - 95.0	63.38	51.1 - 74.5	19/23,(82.6%)	44/71,(62.0%)
Validation Phase	%fPSA	0.16	61.54	40.6 - 79.8	55.56	42.5 - 68.1	16/26,(61.5%)	35/63,(55.5%)
	MALAT-1 Score	95	65.38	44.3 - 82.8	66.67	53.7 - 78.0	17/26,(65.4%)	43/63,(68.3%)

MALAT-1 = metastasis associated in lung adenocarcinoma transcript 1; %fPSA = percent free PSA; PCa = prostate cancer.

Extensive efforts have been devoted to identify novel biomarkers to diagnose PCa more accurately and at early stages to predict tumor recurrence and progression. Numerous emerging biomarkers for PCa have been discovered and tested by clinicians recently, most notably, the urinary markers PCA3 and TMPRSS2:ERG [29, 30]. The Progensa PCA3 test was approved by the US Food and Drug Administration in 2012 to aid in the decision of taking repeat prostate biopsies when the patient had one or more negative biopsies previously. The consensus in most publications is that PCA3 can help distinguish between individuals with and without prostate cancer. A high PCA3 score has a high Gleason score and indicates clinically significant cancers [31]. The performance of PCA3 in Chinese populations has not been evaluated. On the basis of prior reports in Caucasian populations, the AUC of PCA3 in patients with grey zone PSA ranges from 0.64-0.69 [32], which is comparable with the AUC of the MALAT-1 score (0.67-0.74) in our study. However, the limitations of PCA3 include the lack of an appropriate cut-off level for clinical practice, and false negative results may miss some PCa, especially aggressive tumors.

However, several limitations to this study must be acknowledged. First, the sample size was limited in both cohorts, although the initial findings were validated in a prospective multicenter cohort. Second, we did not perform a head-to-head comparison of the MALAT-1 score and the PCA3 score in our study, due to the lack of a commercial PCA3 kit in China. Third, we did not compare the performance of the MALAT-1 score with clinical nomograms (e.g., Prostate Cancer Prevention Trial risk calculator-PCPT) to assess the ability of the MALAT-1 score to increase the AUC of the PCPT risk calculator for predicting PCa diagnosis on biopsy. Fourth, we did not look at the value of MALAT-1 in subgroups of patients classified by initial and repeat biopsy. Future large-scale studies are needed to confirm the efficacy of the MALAT-1 score in the diagnosis of prostate cancer.

In conclusion, we have clearly demonstrated that the MALAT-1 score could serve as a noninvasive biomarker for detecting PCa, especially in the PSA 4-10 ng/ml cohort. Applying a probability threshold of 25%, the MALAT-1-based model would prevent 30.2%-46.5% of unnecessary biopsies in the PSA 4–10 ng/ml cohort, while no high-grade cancers would be missed. This study is limited by the small sample size. Further large-scale studies are needed to confirm our findings.

## METHODS

### Patients and clinical specimens

This multicenter Chinese study was approved by the institutional review board of each participating hospital. Informed consent was obtained from all patients. Subjects were recruited between May 2012 and December 2013 at three urology centers in China (Changhai Hospital, Shanghai; Changzheng Hospital, Shanghai; and West China Hospital, Sichuan). All urine samples were collected from patients scheduled for prostate biopsy because of elevated serum PSA levels (≥4 ng/ml) and/or suspicious DRE. Patients with other known tumors and those receiving medical therapy known to affect serum PSA levels and/or previous PCa therapies were excluded from the study.

### Study design

Our study was divided into two phases (Figure 1): (1) Discovery phase. The MALAT-1 score was tested in an independent cohort of urine sediment samples from 218 consecutive patients, including 85 PCa patients (positive prostate biopsies) and 133 patients with negative prostate biopsies in Shanghai Changhai Hospital who underwent a prostate biopsy. The discriminative power of the MALAT-1 score and its association with the PCa detection rate were evaluated. Then, the diagnostic performance and clinical value of the MALAT-1 score were assessed in the overall cohort and in the grey zone cohort (PSA 4-10 ng/ml) samples. (2) Validation phase. The association of the MALAT-1 score and other parameters with the PCa detection rate was validated in 216 samples (81 positive prostate biopsy and 135 negative prostate biopsies) from the Chinese Prostate Cancer Consortium (CPCC) multicenter samples. Then, the diagnostic accuracy of the MALAT-1 score and other parameters was validated.

### Specimen collection and sample preparation

First-catch urine samples were collected following an attentive DRE (three strokes per lobe) before the biopsy was performed. The urine samples were immediately cooled on ice and were processed within two hours of collection. Biopsies were performed using an end-ﬁre ultrasound transducer (Falcon 2101; B-K Medical, Inc.) and an automatic 18-gauge needle (Bard, Inc.). In all subjects, a 10-12 core systematic laterally directed transrectal ultrasound (TRUS)–guided biopsy was performed.

The urine samples were centrifuged at 2,500 × *g* for 15 min at 4°C and the pellets were washed twice with cold PBS (1×). The sediments were then homogenized in TRIzol reagent and were used for RNA extraction or stored at −80°C until further use.

### Quantitative RT-PCR analysis

Total RNA was extracted from the urine sediments using TRIzol reagent (Invitrogen: No 15596-026, USA). Then, 50 ng of total RNA was treated with DNase I (TaKaRa: D2215, TaKaRa, Japan) prior to cDNA synthesis and was amplified using the TransPlex Complete Whole Transcriptome Amplification Kit (WTA2, Sigma-Aldrich, St. Louis, MO, USA) according to the manufacturer's instructions. qRT-PCR was performed using SYBR^®^ Premix Ex Taq™ (Perfect Real Time) (Takara: DRR081A TaKaRa, Japan) with an Applied Biosystems Step One Plus according to the manufacturer's recommended cycling conditions. The gene-specific sequence information for the qRT-PCR primers is listed as follows: PSAKIT-forward primer GTCTGCGGCGGTGTTCTG, PSAKIT-reverse primer TGCCGACCCAGCAAGATC; MALAT-1 forward primer CTTCCCTAGGGGATTTCAGG, MALAT-1 reverse primer GCCCACAGGAACAAGTCCTA. Briefly, 2 μl of the cDNA solution was amplified using 10 μl of SYBR^®^ Premix Ex Taq™ (Perfect Real Time) (2×) (Takara: DRR081A TaKaRa, Japan), 2 μl of primers, 0.4 μl of ROX Reference Dye (50×) and nuclease-free H_2_O at a final volume of 20 μl. The data were analyzed with StepOne Software version v2.1 (Applied BioSystems, USA). A melt-curve analysis was enabled at the end of the amplification. Samples with PSA Ct values of >28 [20] were excluded to ensure sufficient prostate cell collection. The MALAT-1 score was calculated as MALAT-1 mRNA/PSA mRNA×1000=2^Ct(PSA)-Ct(MALAT-1)^×1000. All experiments were performed in triplicate. No amplification of the signal was obtained when nuclease-free water was added instead of cDNA. The data were analyzed using StepOne Software version v2.1 (Applied BioSystems, USA).

### Statistical analysis

The Mann-Whitney *U*-test, Student's *t*-test, Pearson's chi-square test and Fisher's exact test were used for statistical comparisons of continuous and categorical variables as appropriate. Univariate and multivariate logistic regressions were used to identify independent predictors of PCa upon biopsy. Co-relationships between MALAT-1 and the clinical variables were assessed by the Spearman rank correlation test. Receiver operating characteristic (ROC) curves were constructed to discriminate among different groups of patients. The area under the ROC curve (AUC) was used to assess the predictive power. The sensitivity and specificity were calculated according to the standard formulas. Decision curve analysis was used to evaluate the clinical effects of the calculators.

All of the p values were two-sided, and p<0.05 was considered to be statistically significant. All of the statistical calculations were performed using SPSS (Statistical Package for the Social Sciences) v.17.0 (SPSS Inc., Chicago, IL, USA), MedCalc v.10.4.7.0 (MedCalc Software bvba, Mariakerke, Belgium) and R software v.3.1.1 (The R Foundation for Statistical Computing).

## SUPPLEMENTARY MATERIAL FIGURES


