# Supplementary material for: Development and prospective multicenter evaluation of the long noncoding RNA MALAT-1 as a diagnostic urinary biomarker for prostate cancer
Source: Oncotarget. 2014 Nov 4;5(22):11091–102. doi: 10.18632/oncotarget.2691 (PMC4294360; doi:10.18632/oncotarget.2691)
Supplement: Supplementary file 1 [file oncotarget-05-11091-s001.pdf]

Development and prospective multicenter evaluation of the long noncoding RNA MALAT-1 as a diagnostic urinary biomarker for prostate cancer

Supplementary Material

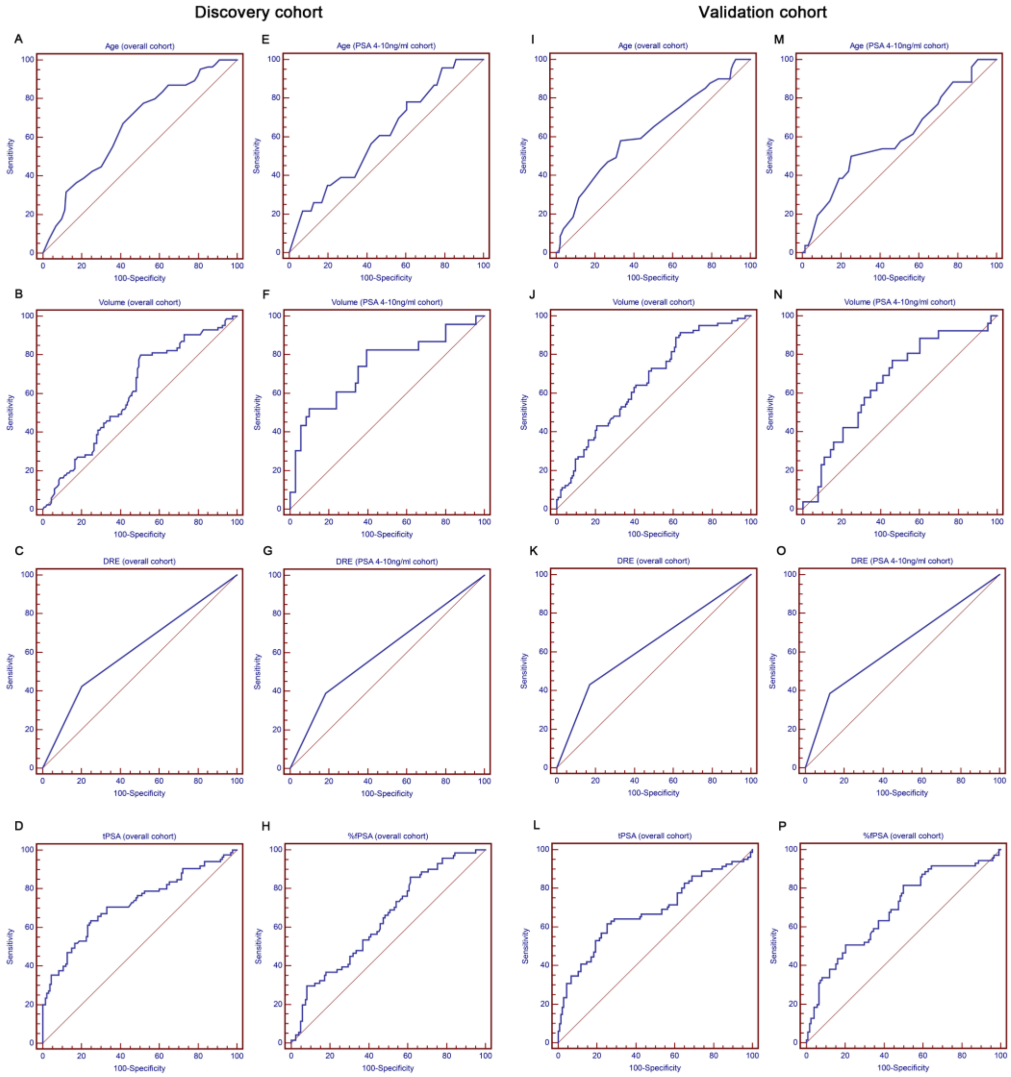

Supplemental Figure 1: Receiver operating characteristic curve analysis for evaluating the diagnostic performance of (A) age, (B) volume (C) DRE, (D) tPSA, (H) %fPSA in the overall cohort in the discovery phase; (E) age, (F) volume (G) DRE in the PSA 4-10 ng/ml cohort in the discovery phase; (I) age, (J) volume (K) DRE, (L) tPSA, (P) %fPSA in the overall cohort in the validation phase; (M) age, (N) volume (O) DRE in the PSA 4-10 ng/ml cohort in the validation phase. PSA=prostate-specific antigen; tPSA=total PSA; %fPSA=percent free PSA; DRE=digital rectal examination.

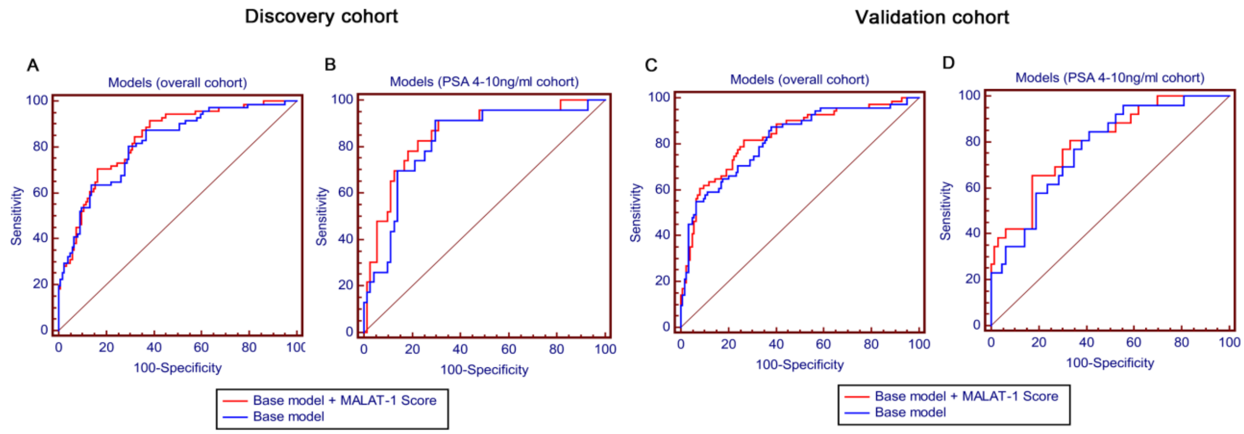

Supplemental Figure 2: Comparison of the diagnostic performance of the base model and the base model plus MALAT-1 in the overall cohort in the (A) discovery phase and (C) validation phase and in the PSA 4-10 ng/ml cohort in the (B) discovery phase and (D) validation phase. The base model in the overall cohort includes age, volume, %fPSA, tPSA and DRE; the base model in the PSA 4-10 ng/ml cohort includes age, volume, %fPSA and DRE.

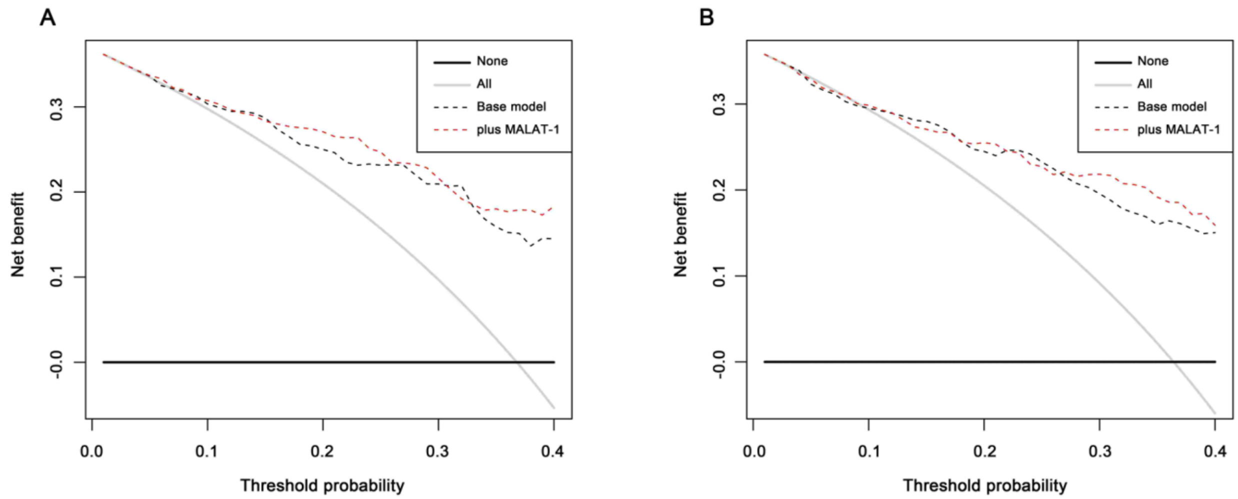

Supplemental Figure 3: Decision curve analysis for positive biopsy prediction in the overall cohort by the base model (base model includes age, volume, %fPSA, tPSA and DRE) in the (A) discovery phase and (B) validation phase. The dashed black line indicates the base model that includes age, volume, %fPSA and DRE; the solid black line shows the prediction model that includes only age, PSA level, DRE result, and prostate volume. The horizontal line along the *x*-axis assumes that no patient will have PCa (no patient should undergo a prostate biopsy), whereas the solid gray line assumes that all patients will have PCa (all patients will need to undergo a prostate biopsy).

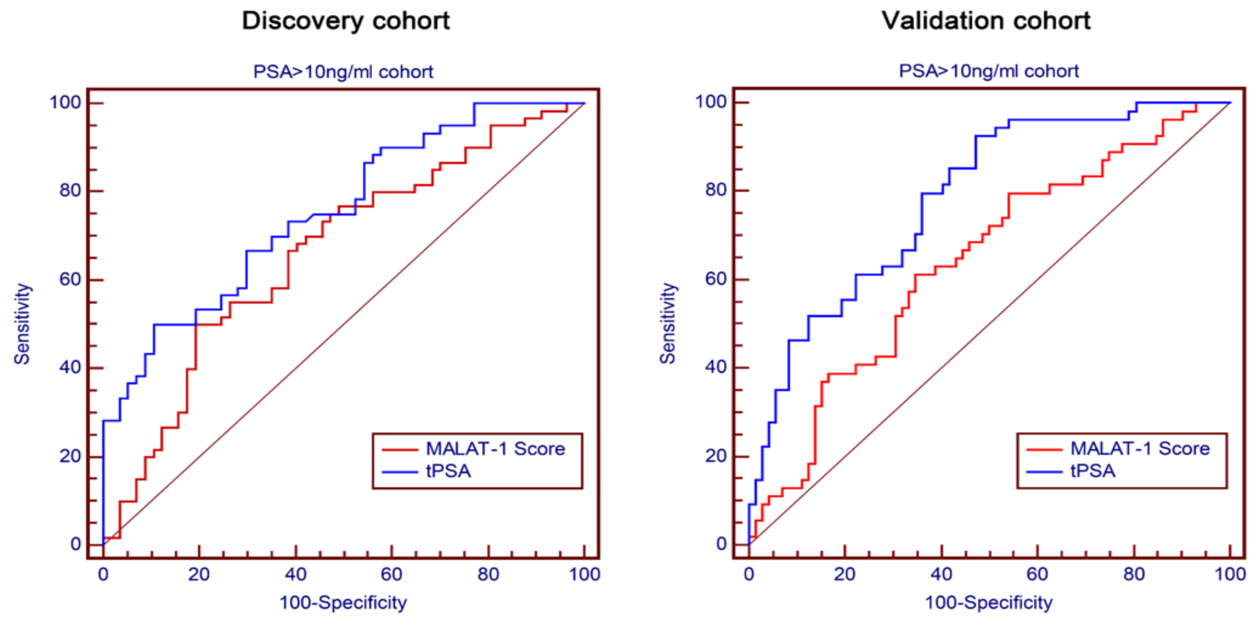

Supplemental Figure 4: Receiver operating characteristic curve analysis for evaluating the diagnostic performance of the MALAT-1 score and PSA in the PSA>10 ng/ml cohort. Comparison of the area under the curve (AUC) of the MALAT-1 score and PSA in the PSA>10 ng/ml cohort in the (A) discovery phase and (B) validation phase.

Supplemental table 1: Co-relationship between MALAT-1 and clinical variables

Variables	MALAT-1 Score at Discovery phase		MALAT-1 Score at Validation phase	
	Correlation Coefficient	*p* value†	Correlation Coefficient	*p* value†
Age	0.069	0.311	0.079	0.249
tPSA	0.096	0.158	0.128	0.052
Volume	0.027	0.693	-0.061	0.375
%fPSA	-0.084	0.218	-0.032	0.643
DRE	-0.014	0.833	0.023	0.741
GS	-0.006	0.959	0.089	0.431

MALAT-1 = metastasis associated in lung adenocarcinoma transcript 1; PSA = prostate-specific antigen; tPSA = total PSA; %fPSA = percent free PSA; DRE = digital rectal examination; GS = Gleason Score.

†Spearman rank correlation test.

Supplemental table 2: Comparison ROC curves between MALAT-1 and clinical variables

Variables	MALAT-1 Score at Discovery phase				MALAT-1 Score at Validation phase			
	Overall Cohort		PSA 4-10ng/ml Cohort		Overall Cohort		PSA 4-10ng/ml Cohort	
	AUC Difference (95%CI)	*p* value[△]	AUC Difference (95%CI)	*p* value[△]	AUC Difference (95%CI)	*p* value[△]	AUC Difference (95%CI)	*p* value[△]
Age	0.0295 (-0.0748-0.134)	0.579	0.131 (-0.0427-0.305)	0.139	0.0364 (-0.0716-0.144)	0.509	0.0693 (-0.110-0.249)	0.449
tPSA	0.0331 (-0.0654-0.132)	0.510	0.197 (0.0151-0.379)	0.034	0.0190 (-0.0868 to 0.125)	0.725	0.0684 (-0.116-0.253)	0.467
Volume	0.0801 (-0.0226-0.183)	0.126	0.00367 (0.0151-0.379)	0.964	0.000503 (-0.103-0.104)	0.992	0.0110 (-0.163-0.185)	0.901
%fPSA	0.0515 (-0.0573- 0.160)	0.353	0.120 (-0.0549-0.295)	0.179	0.0444 (-0.0624-0.151)	0.415	0.0424 (-0.120-0.205)	0.608
DRE	0.0774 (-0.0256-0.181)	0.141	0.137 (-0.0395-0.314)	0.128	0.0303 (-0.0750-0.136)	0.573	0.0409 (-0.135-0.217)	0.648

ROC = receiver operating characteristic; MALAT-1 = metastasis associated in lung adenocarcinoma transcript 1; AUC = area under the ROC curve; PSA = prostate-specific antigen; tPSA = total PSA; %fPSA = percent free PSA; DRE = digital rectal examination; GS = Gleason Score.

[△]Z-Test.

Supplemental table 3: Net benefit and net reduction in avoidable biopsies for the base model and Base model plus MALAT-1 compared to the "treat all" strategy to biopsy every patient for different threshold probabilities in the same range for the overall cohort.

	Threshold probability (%)		10	15	20	25	30	35	40
Discovery Phase	Net benefit	Base model[‡]	30.340	28.711	20.984	23.143	20.947	15.903	14.508
		Base model[‡] plus MALAT-1	30.743	28.315	25.000	24.698	21.614	18.015	18.307
		Treat all	29.764	25.632	27.073	15.717	9.697	2.750	-5.354
	Net reduction in avoidable biopsies	Base model[‡]	5.181	17.444	16.062	22.280	26.252	24.426	29.793
		Base model[‡] plus MALAT-1	8.808	15.199	24.352	26.943	27.807	28.349	35.492
Validation Phase	Net benefit	Base model[‡]	29.516	27.994	24.487	23.248	19.560	15.976	15.043
		Base model[‡] plus MALAT-1	29.858	27.059	25.513	22.735	21.832	19.172	15.897
		Treat all	29.345	25.189	20.513	15.214	9.158	2.170	-5.983
	Net reduction in avoidable biopsies	Base model[‡]	1.538	15.897	15.897	24.103	24.274	25.641	31.538
		Base model[‡] plus MALAT-1	4.615	10.598	20.000	22.564	29.573	31.575	32.821

MALAT-1 = metastasis associated in lung adenocarcinoma transcript 1. PSA = prostate-specific antigen; tPSA = total PSA; %fPSA = percent free PSA; DRE = digital rectal examination;

[‡]The base model consisted of age, tPSA, volume, %fPSA and DRE.

Supplemental table 4: Number of total and high-grade (defined as Gleason ≧ 7) PCa missed and reduction in biopsies according to threshold probability in the range of 10-40% for the base model and base model plus MALAT-1 for patients of the overall cohort.

	Probability cut-off, %	Model	Cancers missed, No. (%)	High-grade cancer missed, No. (%)	Unnecessary Biopsies spared, No. (%)
Discovery Phase	15	Base model‡	1(1.4%)	0	20(16.4%)
		Base model‡ + MALAT1	1(1.4%)	0	18(14.8%)
	20	Base model‡	1(1.4%)	0	19(15.6%)
		Base model‡ + MALAT1	2(2.8%)	0	28(23.0%)
	25	Base model‡	2(2.8%)	0	25(20.5%)
		Base model‡ + MALAT1	2(2.8%)	0	31(25.4%)
	30	Base model‡	2(2.8%)	0	28(23.0%)
		Base model‡ + MALAT1	2(2.8%)	0	32(26.2%)
	35	Base model‡	2(2.8%)	0	30(24.6%)
		Base model‡ + MALAT1	2(2.8%)	0	33(27.0%)
	40	Base model‡	2(2.8%)	0	34(27.9%)
		Base model‡ + MALAT1	2(2.8%)	0	38(31.1%)
Validation Phase	15	Base model‡	2(2.8%)	1(1.4%)	18（14.5%）
		Base model‡ + MALAT1	0	0	13(10.48%)
	20	Base model‡	2(2.8%)	1(1.4%)	18（14.5%）
		Base model‡ + MALAT1	1(1.4%)	0	24(19.4%)
	25	Base model‡	2(2.8%)	1(1.4%)	28(22.6%)
		Base model‡ + MALAT1	1(1.4%)	0	28(22.6%)
	30	Base model‡	2(2.8%)	1(1.4%)	28(23.0%)
		Base model‡ + MALAT1	2(2.8%)	1(1.4%)	35(28.2%)
	35	Base model‡	2(2.8%)	1(1.4%)	30(24.2%)
		Base model‡ + MALAT1	2(2.8%)	1(1.4%)	37(29.8%)
	40	Base model‡	2(2.8%)	1(1.4%)	37(29.8%)
		Base model‡ + MALAT1	2(2.8%)	1(1.4%)	39(31.5%)

PCa = prostate cancer; MALAT-1 = metastasis associated in lung adenocarcinoma transcript 1; PSA = prostate-specific antigen; tPSA = total PSA; %fPSA = percent free PSA; DRE = digital rectal examination;

‡The Base model consisted of age, tPSA, volume, %fPSA and DRE.
